# Iron Deficiency Anemia and Migraine: A Literature Review of the Prevalence, Pathophysiology, and Therapeutic Potential

**DOI:** 10.7759/cureus.69652

**Published:** 2024-09-18

**Authors:** Zahraa M Al-Qassab, Osman Ahmed, Vaishnavi Kannan, Najeeb Ullah, Sunitha Geddada, Amir T Ibrahiam, Marcellina Nwosu

**Affiliations:** 1 Internal Medicine, California Institute of Behavioral Neurosciences & Psychology, Fairfield, USA; 2 General Surgery Research, California Institute of Behavioral Neurosciences & Psychology, Fairfield, USA; 3 General Surgery, California Institute of Behavioral Neurosciences & Psychology, Fairfield, USA; 4 Clinical Research, California Institute of Behavioral Neurosciences & Psychology, Fairfield, USA

**Keywords:** iron deficiency anemia (ida), iron therapy, menstrual migraine, migraine disorder, serum ferritin

## Abstract

This literature review explores the association between iron deficiency anemia and migraines, examining their prevalence, underlying pathophysiology, and therapeutic potential of iron supplementation in managing migraine symptoms. A systematic search across major academic databases, including PubMed, Google Scholar, ScienceDirect, and Science.gov, identified relevant peer-reviewed studies that focus on the link between iron deficiency anemia and migraines. The review highlights a higher prevalence of iron deficiency anemia among migraine sufferers, particularly women, with several studies indicating a significant inverse relationship between serum ferritin levels and migraine severity. The findings suggest that low iron levels may exacerbate migraine symptoms, and iron supplementation has been shown to reduce the frequency and intensity of migraines, especially in patients with confirmed iron deficiency anemia. The review also discusses the unique susceptibility of women, particularly those of reproductive age, to both iron deficiency anemia and migraines, emphasizing the importance of gender-specific approaches in treatment. Moreover, the potential impact of iron deficiency on neurovascular function and its contribution to migraine pathogenesis is explored, reinforcing the need for comprehensive management strategies that include nutritional interventions. While iron supplementation appears to be a promising therapeutic option, further research is required to fully understand its long-term effects and to refine treatment protocols to avoid the risks associated with iron overload. This review underscores the significance of addressing nutritional deficiencies as part of a holistic approach to migraine management, offering insights into potential avenues for improving patient outcomes.

## Introduction and background

Migraine is a common neurological disorder that is characterized by recurrent, often severe, headaches. They are frequently accompanied by nausea, photophobia, and phonophobia, significantly impacting the quality of life for sufferers [1]. Migraine affects around 12% of the adult population in the world. The prevalence is notably higher in women, at approximately 18%, compared to 6% in men [2]. Moreover, it has been established that migraine is particularly more prevalent in women during their reproductive years [3]. It is also the second leading cause of disability globally, having the potential to inflict personal, social, and economic burdens on affected individuals [4], as well as having a major impact on sleep, cardiovascular, and psychiatric health [5]. The pathophysiology of migraine is multifactorial with an interplay between genetic and environmental factors [6,7]. One area of upcoming research is increasingly focusing on the role of nutritional deficiencies, particularly iron deficiency, as a potential contributor to the development of migraine.

Iron deficiency is the most common nutritional deficiency, affecting approximately 25% of the global population [8]. Women of childbearing age are especially at risk for this condition with 10% compared to 1% in men [9]. Iron plays a vital role in various physiological processes that support brain functions and overall neurological health such as oxygen transport, DNA synthesis, and energy metabolism [10].

Many studies have pointed out the possible link between iron deficiency and migraine, especially in adult women. A study by Singh et al. showed that chronic daily headaches in adults were significantly related to iron-deficiency anemia (IDA), suggesting that severe forms of IDA might exacerbate migraines' severity [11]. Another study by Rashid et al. concluded that 53.1% of young females who suffer from migraines were diagnosed with IDA, with menstrual abnormalities showing a statistically significant association with the presence of anemia [12].

Tayyebi et al. further demonstrated that hemoglobin and serum ferritin levels were significantly associated with the occurrence of migraines in women, suggesting that low iron stores could be one of the causes of migraine incidence [13]. This correlation is supported by additional research showing that iron deficiency can impair neurovascular function - an event critical in the pathogenesis of migraine attacks [14]. There could be a potential connection to IDA, which offers a novel therapeutic approach for migraine management [15].

The relationship between migraines and iron deficiency, particularly in adult women, highlights the critical role of nutritional health in managing these conditions. Addressing iron deficiency through appropriate dietary supplementation may not only reduce the frequency and severity of migraine attacks but also enhance overall health outcomes. This approach emphasizes the interconnected nature of physiological systems, illustrating that diet and nutrition can significantly impact chronic conditions such as migraines.

## Review

Methodology

This literature review employed a systematic search across four major academic databases - PubMed, Google Scholar, ScienceDirect, and Science.gov - to identify peer-reviewed articles examining the association between migraine and iron deficiency. The review focused on assessing the prevalence of iron deficiency among adults with migraines, exploring the mechanisms linking iron deficiency to migraine pathophysiology, evaluating the impact of iron supplementation on migraine outcomes, and identifying at-risk subpopulations, particularly women of reproductive age. A comprehensive search strategy using relevant keywords and MeSH terms with keywords such as "migraine," "iron deficiency," "iron deficiency anemia," "prevalence," "supplementation," and "neurological impact" was used. The inclusion criteria focused on studies involving adults, peer-reviewed original research, and reviews published in English. Data extraction centered on study design, sample size, demographics, prevalence rates, and outcomes related to iron supplementation. The synthesized findings provide insights into the role of iron deficiency in migraines, with implications for clinical practice and potential therapeutic strategies.

Results

The studies reviewed collectively suggest a significant connection between IDA and the occurrence and severity of migraines, particularly among women. These studies generally show a higher prevalence of IDA in migraine sufferers compared to control groups, with some research pointing out that this is especially true for menstrual migraines (MM) [16]. Strong statistical associations have been found, such as a p-value of 0.002 linking low dietary iron intake to severe headaches or migraines [17], and a p-value of 0.018 connecting IDA with MM [16]. Studies have consistently shown an inverse relationship between ferritin levels and migraine severity. Lower ferritin levels are associated with higher visual analog scale (VAS) scores (p=0.006) and headache impact test (HIT-6) scores (p=0.01) [18]. While not all studies have established a direct relationship between the severity of anemia and the frequency of migraines across different subgroups, there is clear evidence that iron deficiency, especially low ferritin levels, is linked to more severe migraine symptoms [13]. Moreover, it is reported that women are more frequently affected by both migraines and IDA, highlighting the need for gender-specific considerations in future research [17]. Table 1 summarizes the relevant studies, highlighting interventions, results, and conclusions.

**Table 1:** Summary of relevant studies IDA: Iron Deficiency Anemia; NHANES: National Health and Nutrition Examination Surveys; VAS: Visual Analog Scale; HIT-6: Headache Impact Test; MM: Menstrual Migraine; TTH: Tension-Type Headache

Author and Year of Publication	Study Design	Intervention	Results	Mean Age	Male-to-Female Ratio	Conclusion	Comments
Mohammadi et al. (2016) [19]	Descriptive-analytic study	Investigation of the association between IDA and MM in women.	No significant relationship between IDA and MM (p=0.18). Significant association between IDA and migraine with aura (p=0.04).	34.5 years	Only female participants	Although IDA was more prevalent among patients with MM, it was not significantly associated with MM.	Small sample size, lack of representation of the general population.
Tayyebi et al. (2019) [13]	Case-control study	Assessment of the relationship between IDA and migraine in both male and female patients.	Significant differences in hemoglobin, serum ferritin levels, and IDA between female cases and controls (p=0.0004, p=0.006, p=0.001). No significant differences among males (p>0.05).	37.63 years (case group), 34.93 years (control group)	24 males and 76 females in both case and control groups	The study suggests an association between IDA and the incidence of migraines, particularly in females. Iron supplements might be effective in treating migraines associated with IDA.	Small sample size of male participants, and the study was conducted in a specific clinical setting, which may limit generalizability.
Meng et al. (2021) [17]	Cross-sectional study using data from the National Health and NHANES	Assessment of the association between dietary iron intake, serum ferritin levels, and the prevalence of severe headache or migraine among American adults.	Dietary iron intake was inversely associated with severe headache or migraine in women aged 20–50 years (p = 0.002). Serum ferritin was negatively associated with severe headache or migraine in women over 50 years (p < 0.001). No significant association between dietary iron intake or serum ferritin and migraines in men.	51 years (control), 44 years (cases)	49.8% male, 50.2% female (controls) 29.1% male, 70.9% female (cases)	The study suggests that increasing dietary iron intake may help prevent severe headaches or migraines in women aged 20-50 years, while higher serum ferritin levels may have a protective effect in women over 50 years.	Limitations include reliance on self-reported data for headache diagnosis, potential inaccuracies in dietary recall, and the inability to establish causality due to the cross-sectional design.
Sari et al. (2024) [18]	Retrospective cross-sectional study	Investigation of the effect of anemia severity and iron parameters on the frequency and severity of migraine attacks.	No significant difference in migraine frequency and severity across anemia subgroups. MM was associated with low hemoglobin levels (p = 0.03). Significant increase in VAS (p = 0.006) and HIT-6 scores (p = 0.01) with low ferritin levels.	32 patients with mild anemia: 36.6 ± 12.5 years 41 patients with moderate anemia: 46 ± 14.2 years 31 patients with severe anemia: 52.4 ± 13.7 years	85.6% female, 14.6% male	The presence and severity of anemia and iron deficiency do not affect migraine frequency and severity in general, but an inverse relationship was found between VAS, HIT-6, and ferritin levels. VAS was found to be more effective than HIT-6 in reflecting migraine severity.	Limitations included a small sample size and a predominantly female study population. Further research is needed to confirm the findings and explore gender differences.
Gür-Özmen et al. (2016) [16]	Case-control study	Investigation of the association between IDA and different types of headaches, including MM.	IDA was more common in migraine patients compared to controls (21.7% vs 12.9%, p=0.02). A significant association between IDA and MM (p=0.018).	35.05 years	85.6% female, 14.6% male	The study suggests a significant association between IDA and MM, particularly in women. The study highlights the potential role of estrogen, iron metabolism, and dopamine dysfunction in the pathophysiology of menstrual migraines.	Limitations include the use of a hospital-based control group, which is not representative of the general population.

 Discussion

Prevalence of Iron Deficiency Anemia in Migraine Patients

The prevalence of IDA among migraine patients has emerged as a significant focus in recent research. Studies have consistently demonstrated a higher prevalence of IDA in individuals suffering from migraines compared to the general population. For instance, Gür-Özmen et al. reported that approximately 26.3% of patients with MM exhibited IDA, a notable contrast to the 20% observed in non-MM patients [19]. Similarly, Tayyebi et al. found that 22% of female migraine patients were diagnosed with IDA, compared to only 3.9% in the control group [13]. These findings suggest a compelling link between migraine and iron deficiency, particularly among women.

The relationship between IDA and migraine could be attributed to multiple factors, including hormonal changes, nutritional deficiencies, or even genetic predispositions [6,7]. Our analysis suggests that the prevalence of IDA among migraine sufferers, particularly females, may be underreported due to variations in diagnostic criteria and population differences across studies. Nevertheless, several studies consistently highlight higher rates of IDA in patients with migraines, which justifies the need for routine assessment of iron levels in this population. Implementing such measures could facilitate the accurate identification and treatment of IDA, potentially reducing both the frequency and intensity of migraine attacks through adequate iron supplementation.

Impact of Iron Deficiency on Migraine Frequency and Severity

Recently, various studies have been conducted on the significance of iron deficiency in terms of the frequency and severity of migraine. For instance, a study by Sari et al. found that, while anemia severity did not universally impact migraine frequency and severity, an inverse relationship was observed between ferritin levels and the severity of migraines as measured by the VAS and HIT-6 scores. Table 2 illustrates the relationship between the severity and frequency of migraine attacks and IDA. Low ferritin, a sign of iron deficiency, could be correlated with more severe presentations of migraine, particularly those related to menstruation [18].

**Table 2:** The relationship between severity and frequency of migraine attacks and iron deficiency anemia TTH: Tension-Type Headache; HIT-6: Headache Impact Test-6; VAS: Visual Analogue Scale; HAM-A: Hamilton Anxiety Rating Scale; BMI: Body Mass Index; TIBC:Total Iron-Binding Capacity; Rbc: Red Blood Cell; MCV: Mean Cell Volume; RDW: Red Cell Distribution Width [18]

Demographic Variables	Without Anemia	Anemia	p (<0.05)	Ferritin ≥ 20 ng/mL	Ferritin < 20 ng/mL	p (<0.05)
Headache			0.60			0.23
With Aura	2	2		1	0	
Without Aura	31	24		36	29	
Aura+TTH	3	4		0	0	
Without Aura+TTH	2	2		4	2	
Menstrual Migraine	5	42	0.02	4	43	0.015
Headache Frequency	5.47 ± 3.75	6.40 ± 4.16	0.34	5.53 ± 2.91	5.70 ± 3.65	
N (Mean)						
HIT-6	25.2 ± 0.6	25.6 ± 0.6	0.74	47.7 ± 2.1	55.0 ± 1.1	0.006
VAS	5.8 ± 0.2	5.8 ± 0.2	0.97	5.1 ± 0.1	6.1 ± 0.1	0.000
HAM-A	50.2 ± 2.1	52.7 ± 2.2	0.086	2.6 ± 0.1	2.8 ± 0.1	0.61
BMI	2.7 ± 0.1	2.2 ± 0.2	0.35	23.9 ± 0.4	25.9 ± 0.7	0.32
Iron µg/dl	69.2 ± 4.2	41.4 ± 4.7	0.00	25.7 ± 2.0	22.2 ± 1.8	0.00
TIBC	259.5 ± 9.6	372.2 ± 11.9	0.00	393.8 ± 13.0	408.6 ± 9.7	0.00
Ferritin	28.3 ± 3.2	13.6 ± 2.0	0.00	14.3 ± 3.8	10.4 ± 1.0	0.00
Hemoglobin (g/dL)	13.5 ± 1.06	11.5 ± 0.3	0.00	9.5 ± 0.7	7.2 ± 0.8	0.00
Rbc	4.5 ± 0.05	4.5 ± 0.1	0.006	4.1 ± 0.05	4.1 ± 0.01	0.02
MCV	79.1 ± 0.7	79.4 ± 0.6	0.79	77.8 ± 0.4	77.5 ± 1.0	0.19
RDW	12.1 ± 0.3	16.4 ± 0.1	0.00	17.6 ± 0.09	16.7 ± 0.37	0.00

Another study by Singh et al. demonstrated a significant association between chronic daily headache severity and IDA. The study found that patients with severe IDA had more frequent and severe headaches, suggesting that iron deficiency may exacerbate migraine symptoms. This highlights the need to assess and address nutritional deficiencies, particularly iron, in chronic migraine patients. Moreover, correcting iron deficiency may lead to a reduction in both the frequency and intensity of migraines [11].

Other studies support these findings, demonstrating that iron supplementation is effective in reducing both the intensity and frequency of migraines in patients with iron deficiency. For example, Fallah et al. proved that the use of iron therapy with ferrous sulfate significantly reduced monthly frequency, severity, duration, and disability scores of headaches in children with migraines, suggesting similar benefits could be expected in adults. Table 3 illustrates the comparison of headache characteristics before and after iron therapy [20]. Overall, these findings suggest a potential link between iron deficiency and migraine attacks, as iron supplementation alleviated and reduced both the frequency and severity of these attacks.

**Table 3:** Comparison of headache characteristics before and after iron therapy Ref. [20]

Variables	Before treatment	After treatment	p-value
Monthly headache frequency	22.89 ± 7.18	10.13 ± 4.51	0.001
Headache duration in hours	2.14 ± 1.23	1.14 ± 1.01	0.001
Severity score of headache	8.12 ± 1.76	5.11 ± 1.62	0.001
Headache disability score	38.23 ± 10.7	22.87 ± 8.65	0.01

MM and Iron Deficiency Anemia

Several studies have documented the significance of the relationship between MM and IDA. Gür-Özmen et al. reported a significant association between IDA and both pure MM (PMM) and menstrually related migraine (MRM). In this case-control study, IDA was found to be significantly more prevalent in women with these types of migraines compared to those whose migraines were not related to their menstrual cycle [19].

Similarly, Vukovic-Cvetkovic et al. reported that IDA was significantly more common in women with PMM and MRM. Their post hoc analysis revealed that women experiencing migraines closely tied to their menstrual cycles had a higher likelihood of IDA, further solidifying the relationship between these conditions [21].

Moreover, Rashid et al. identified menstrual abnormalities as a key factor associated with the presence of IDA in young female migraine patients. Their findings suggest that the hormonal fluctuations and blood loss associated with menstruation may exacerbate iron deficiency, leading to an increased incidence of migraines in this population [12].

These studies collectively highlight the importance of monitoring and addressing iron levels in women suffering from MM. This strategy for managing iron deficiency, especially in patients with heavy menstrual bleeding, could be vital for reducing both the frequency and severity of MM. It also offers a more targeted approach to migraine management within this specific subgroup.

Neurovascular Function and Iron Deficiency in Migraine Pathogenesis

Neurovascular function appears to have an important role in the pathogenesis of migraine, particularly in states of iron deficiency. IDA has been shown to impair neurovascular function, which could be a contributing factor in migraine development. A study by Suriany et al. demonstrated that IDA in otherwise healthy women was associated with impaired neurovascular function, as evidenced by brain MRI and cognitive testing. This supports the idea that an iron deficiency state can influence cerebrovascular health, which could subsequently trigger or exacerbate a migraine attack and perhaps even cause it [14].

Furthermore, research by Isasi et al. on infant rats revealed that gestational and lactational iron deficiency anemia impairs myelination and the neurovascular unit, indicating that iron plays a critical role in maintaining neurovascular integrity. Although this study was conducted on animals, it provides insights into how iron deficiency could affect neurovascular function in humans, potentially contributing to migraine pathogenesis [22].

Similarly, iron deficiency has been identified as a risk factor for ischemic stroke in patients with pulmonary arteriovenous malformations, as pointed out by Shovlin [23]. This points to a much greater implication of iron deficiency in neurovascular dysfunction that might be relevant to the understanding of its role in migraine pathogenesis [23].

These studies collectively highlight the potential impact of iron deficiency on neurovascular function, suggesting that iron deficiency may contribute to the pathogenesis of migraines by impairing cerebrovascular health and function. Addressing iron deficiency could therefore be a crucial component in managing migraine symptoms and preventing more severe neurovascular complications.

Gender Differences in the Relationship Between Iron Deficiency and Migraine

Gender differences in the relationship between iron deficiency and migraine have been observed in several studies, highlighting how biological and physiological factors may influence the prevalence and severity of migraines differently in men and women. According to Tayyebi et al. [13], a statistically significant association exists between IDA and migraine in females. Differences in hemoglobin and serum ferritin levels suggest a higher prevalence of iron deficiency among women suffering from migraines compared to men. The study shows that the prevalence of IDA among females with migraines is 22%, compared to 3.9% in controls, indicating a statistically significant difference between the two groups (p=0.001) [13].

In addition, Sharma et al. illustrated that oxidative stress in IDA is more pronounced in women than in men. Their study showed that women exhibited higher levels of malondialdehyde (MDA) and lower levels of superoxide dismutase (SOD) and total antioxidant capacity (TAC) than men. This may contribute to more severe migraine pathogenesis in women, possibly due to less efficient compensatory mechanisms in response to iron deficiency [24].

Additionally, Wei et al. observed that women generally have significantly higher rates of inadequate intake of essential nutrients, including iron, compared to men. This nutritional disparity could contribute to the gender differences observed in the prevalence and severity of iron deficiency-related migraines, as women are more likely to experience micronutrient deficiencies that exacerbate migraine symptoms [25].

These findings underscore the importance of considering gender-specific factors when addressing iron deficiency in the context of migraine treatment and prevention. Women, especially those of reproductive age, may benefit from more targeted nutritional interventions to alleviate the impact of iron deficiency on the frequency and severity of migraines.

Therapeutic Implications: Iron Supplementation for Migraine Management

The therapeutic implications of iron supplementation in the management of migraine have gained increasing attention, particularly in light of the established connection between iron deficiency and migraine pathogenesis. Research suggests that addressing iron deficiency through supplementation could be a crucial strategy in alleviating migraine symptoms, especially in individuals with confirmed IDA.

Iron supplementation is considered to be an integral part of the management of IDA, having long been associated with the frequency and intensity of migraines. Pasini et al. also reported that appropriate iron supplementation should not only look at addressing iron sufficiency but should also critically look at the availability of precursor molecules necessary for heme synthesis, including essential amino acids and vitamins. This comprehensive approach is necessary to promote the synthesis of heme and other fundamental enzymes required for optimal metabolism, which may help reduce migraine attacks [26].

Rosignoli et al. highlighted the multifactorial origin of migraines and the potential role of iron supplementation as part of a broader, integrative treatment plan. Their study suggests that combining iron supplementation with other non-pharmacological approaches, such as lifestyle modifications and dietary interventions, could enhance the overall management of migraines, particularly for patients with underlying metabolic abnormalities [27].

Moreover, Hassan et al. [18] explored the therapeutic implications of altered energy metabolism in migraine patients, noting that iron supplementation could be particularly beneficial in addressing the metabolic imbalances that often accompany migraine disorders. By restoring normal iron levels, patients may experience a reduction in the frequency and severity of migraines, leading to improved quality of life [28].

In conclusion, iron supplementation appears to be a promising therapeutic option for managing migraines, especially in patients with documented iron deficiency. By addressing both iron levels and related metabolic pathways, supplementation can potentially reduce migraine symptoms and improve patient quality of life. However, careful monitoring is required to prevent the risks of iron overload, ensuring appropriate supplementation pertaining to individual needs and integrated support treatments.

Challenges and limitations

This literature review provides valuable insights, but it is important to recognize limitations that could affect how findings are interpreted. The studies included in this review vary widely in their design, with many relying on case-control and cross-sectional designs. These types of studies limit the ability to find a causal link between IDA and migraines, as it is not established whether IDA causes migraines or if it's a result of other factors associated with migraines, such as changes in diet or menstrual irregularities. Additionally, the research primarily focuses on women of reproductive age, which means the findings may not apply to other groups, such as men, children, or postmenopausal women. Future studies should include a more diverse range of participants to better understand the broader implications. Finally, while iron supplementation appears to be a promising treatment option, we do not have a proper understanding of its long-term safety and effectiveness. There is a risk of iron overload, which needs to be carefully considered in future research. Addressing these limitations will be crucial for deepening our understanding of the connection between iron deficiency anemia and migraines, and for developing safe, effective treatments based on solid evidence.

## Conclusions

IDA is strongly associated with increased prevalence, frequency, and severity of migraines, especially in women. By addressing the underlying metabolic imbalances linked to IDA, appropriate iron supplementation has shown promising results in relieving migraine symptoms. A holistic and comprehensive treatment approach, including nutritional management and non-pharmacological interventions, could significantly improve patient outcomes.
